# Cancer Trends in West Bengal Over 25 Years: A Comprehensive Single-Centre Study

**DOI:** 10.1007/s13193-025-02252-5

**Published:** 2025-03-01

**Authors:** Shazia Absar, Samir Bhattacharyya, Arnab Gupta

**Affiliations:** 1 School of Clinical Medicine, University of Cambridge, Cambridge, UK; 2 Saroj Gupta Cancer Centre and Research Institute, Mahatma Gandhi Rd, Thakurpukur, Kolkata India

**Keywords:** Cancer, West Bengal, Kolkata, Epidemiology, Breast, Cervical, Lifestyle, Tobacco

## Abstract

The last 25 years have seen considerable changes in cancer epidemiology in India. Numerous studies have shown increases in certain cancers, such as lifestyle and tobacco-related cancers, with decreases in other cancers, such as cervical cancer. Despite the development of the National Cancer Registry Program in 1964, cancer is not a notifiable disease in India, and there remains no comprehensive cancer registry with data relying on regional and hospital-based registries. This retrospective study aimed to elucidate the trends in cancer epidemiology in the West Bengal region over a period of 25 years (1996–2020). Our analysis includes retrospective data from the hospital electronic records of a tertiary cancer institution in Kolkata which serves patients from Kolkata as well as Eastern, North-Eastern India and Bangladesh. We collected data from 189,485 patients (of which 135,578 had malignant disease) who were diagnosed during this time. Data was visualised using Microsoft Excel and analysed using linear trend analyses and the Mann–Kendall test. We saw significant increases in breast cancers amongst females and oral cavity cancers amongst males, consistent with other studies across various regions of India. In contrast, we identified significant decreases in the frequency of cervical cancers amongst females and cancers of the ear, nose and throat regions amongst males. We report that the cancer epidemiology in West Bengal is generally following that of the rest of India. Our study has revealed important trends in cancer frequency in West Bengal and has illustrated important areas for targeting preventative and/or screening interventions.

**Supplementary Information:**

The online version contains supplementary material available at 10.1007/s13193-025-02252-5.

## Introduction

Cancer remains a significant public health challenge in India, with a steadily increasing burden of disease over recent years. In 2022, the projected number of new cancer cases was 1,461,427 with a crude incidence rate of 100.4 per 100,000 individuals [1]. Lung cancer was found to be the most common cancer amongst males whilst breast cancer was most common amongst females [1]. Furthermore, amongst children of both sexes, lymphoid leukaemia was the most common cancer diagnosis, comprising 29.2% of cases in boys and 24.2% in girls [1].

Recent years have seen changes in the incidence of cancer in India in line with that of developed countries. There has been evidence of an increase in the incidence of some cancers, such as breast and colon cancers, whilst other cancers have been shown to be decreasing in incidence, such as cervical cancer [2–6].

Many factors have been implicated in the changes in cancer incidence that have been seen, most notably the urbanisation and modernisation of India which has led to changes in diet as well as increases in sedentary lifestyles and tobacco use. Additionally, changes in cultural norms, such as child marriage and improved education, have been implicated in decreases in cancer incidence seen, particularly for cervical cancer.

Cancer registration in India commenced in 1964 and was subsequently expanded in 1982 with the creation of the National Cancer Registry Program (NCRP) by the Indian Council of Medical Research (ICMR) [7]. The NCRP is a collaboration between the ICMR and various state and central government health agencies with the primary goal of collecting, analysing and disseminating information related to cancer incidence and prevalence in India. Several reports from the NCRP using different registries across India have been published with the most recent comprehensive report being published in 2023 [3]. This report highlighted two key findings. Firstly, the report showed a relatively stable pattern of overall cancer incidence across the country, except for in Delhi, where a rising trend was observed. Secondly, the report highlighted the rising incidence of breast cancer and concurrent decreasing incidence of cervical cancer. However, despite the creation of the NCRP in 1982, this latest report included a mere 38 population-based registries and 184 hospital-based registries [3].

Previous studies have investigated changes in trends of cancer incidence using data from the population registries of specific regions and/or hospital databases [9–20]. Furthermore, there has been previous work specifically investigating trends in West Bengal and/or Eastern India [21, 22].

In our study, we used data from the cancer registry of a tertiary cancer institution in the city of Kolkata, the capital of the Indian state of West Bengal. This hospital serves a diverse population from both Kolkata and its adjacent regions, and during the time period studied, it was one of the two premier comprehensive cancer centres in the region catering to patients from Eastern and North-eastern India as well as from Bangladesh. As a charitable hospital, the population served includes individuals from diverse socio-economic backgrounds, and hence, the trends we found may reliably reflect the whole region. To our knowledge, this is one of the largest single-centre studies analysing time trends in different cancers coming out of India.

In this paper, we used data from the hospital cancer registry to investigate changes in the frequency of diagnosis of different cancer types within the state of West Bengal over a 25-year period between 1996 and 2020, adding to the literature investigating the demographical changes in cancer incidence in Eastern India, and how it compares with the rest of India.

## Materials and Methods

The aim of our study was to analyse trends in the diagnosis of different cancers in a tertiary cancer centre in Eastern India over a 25-year period (1996–2020). We collected data on all cancer patients diagnosed and/or treated in the centre during this period and examined the relative frequencies of the various cancer types across different time intervals. The analysis was conducted for the overall patient population, as well as separately for males and females.

Currently, clinical data at the hospital is recorded electronically. However, in the past, data was collected manually and recorded in the hospital’s records section by data entry operators. All historical manual records have since been digitized and integrated into the electronic database.

We accessed data from the hospital’s cancer database between the years of 1996 and 2020. This included the data from 189,485 patients, of which 135,578 had malignant disease.

The cancer database in the hospital broadly assigned each patient to one of the following groups depending on the region of their cancer diagnosis: head and neck (separated into oral cavity; ear, nose and throat region [external and internal ear, nasal cavity, oropharynx, larynx and pharynx]; and salivary glands), respiratory (separated into lung, trachea and bronchus and pleura, thymus and intra-thoracic organs), digestive system (separated into oesophagus; stomach; duodenum small intestine; peritoneum, colon, rectum and anal canal; liver and gallbladder; and pancreas), bone and connective tissues, skin, female genital system (separated into uterus, cervix, ovary and fallopian tubes, vulva and vagina and placenta), male genital system (separated into testis, penis and prostate), urinary system (separated into kidney, ureter, bladder, urethra and other), thyroid and endocrine, neurology (separated into brain, eye and nervous system), haematology (separated into haematopoietic and lymphatic system including leukaemia, Hodgkin’s disease, non-Hodgkin’s lymphoma and others), unknown (separated into secondary malignant neoplasm from unknown primary and other unspecified sites) and non-malignant and undiagnosed conditions.

Non-malignant and undiagnosed diseases were excluded from the final analysis and the percentage of total malignancies that a particular cancer subtype constitutes was calculated.

These calculations were done for all patients as well as males and females separately. Since 2010, the cancer database automatically assigns those who identify as a different non-binary gender as male so these individuals were included in our analysis under male. This was relevant for 30 individuals, of which 15 had malignant disease, until the year 2010 after which they were automatically included under male.

Data was inputted and visualised using Microsoft Excel.

### Statistical Analysis

Following visualisation, linear trend analysis was conducted to determine the significance of observed changes seen on visualisation [23] (see Supplementary Information).

*R*-squared values were used to test for the applicability of the linear model (range 0–1; 0 = no fit, 1 = perfect fit).

Statistical significance of the constant *b* was tested with *t*-test; a *p* value < 0.05 was considered to be significant.

The non-parametric statistical test Mann–Kendall test was employed to evaluate the presence of a monotonic trend in the variable over time for trends not conforming to the linear model [24]. A positive value for τ indicates an increasing trend over time whilst a negative τ indicates a decreasing trend. Furthermore, a magnitude of τ near zero indicated a weak trend, whilst values nearer 1 or − 1 indicated a stronger trend. The significance of the trends was tested with a *p* value of < 0.05 considered significant.

## Results

A total of 189,485 patients seen at the hospital were included in this study. This included 103,233 patients classified as males and 86,252 patients classified as females. A total of 53,907 patients were excluded from the final analysis as they were classified as having non-malignant or undiagnosed disease. The remaining 135,578 patients were included in the study.

**Fig. 1:**
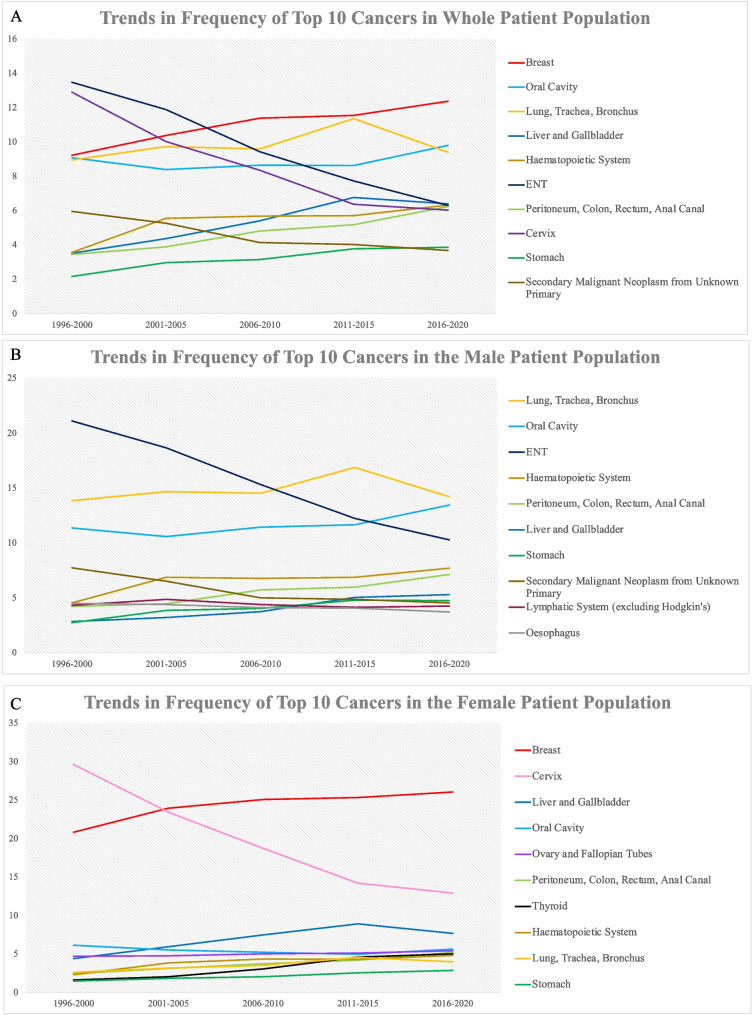
Graph showing trends in the frequency of the top ten cancers in the whole patient population (**A**), male patient population (**B**) and female patient population (**C**). Data is presented as a percentage of the total number of malignant cases for that time period

The top ten most common cancers amongst the whole population in the most recent period (2016–2020) were breast cancers, followed by oral cavity cancers; lung, trachea and bronchus cancers; liver and gallbladder cancers; cancers of the haematopoietic system; cancers of the ear, nose and throat regions; peritoneum, colon, rectum and anal canal cancers; cervical cancer; stomach cancer; and secondary malignant neoplasm from unknown primary. This differed from the trend amongst patients classified as male where the most common cancers in the most recent period were lung, trachea and bronchus cancers, followed by oral cavity cancers; cancers of the ear, nose and throat regions; cancers of the haematopoietic system; peritoneum, colon, rectum and anal canal cancers; liver and gallbladder cancers; stomach cancer; secondary malignant neoplasm from unknown primary; lymphatic system cancers (excluding Hodgkin’s); and oesophageal cancers. The trend amongst females was also highly distinct with the most common cancer being breast cancers, followed by cervical cancer; liver and gallbladder cancers; oral cavity cancers; ovarian and fallopian tube cancers; peritoneum, colon, rectum and anal canal cancers; thyroid cancers; cancers of the haematopoietic system; lung, trachea and bronchus cancers; stomach cancer; secondary malignant neoplasm from unknown primary; and uterine cancers (Fig. 1 and Supplementary Table 1).

**Fig. 2:**
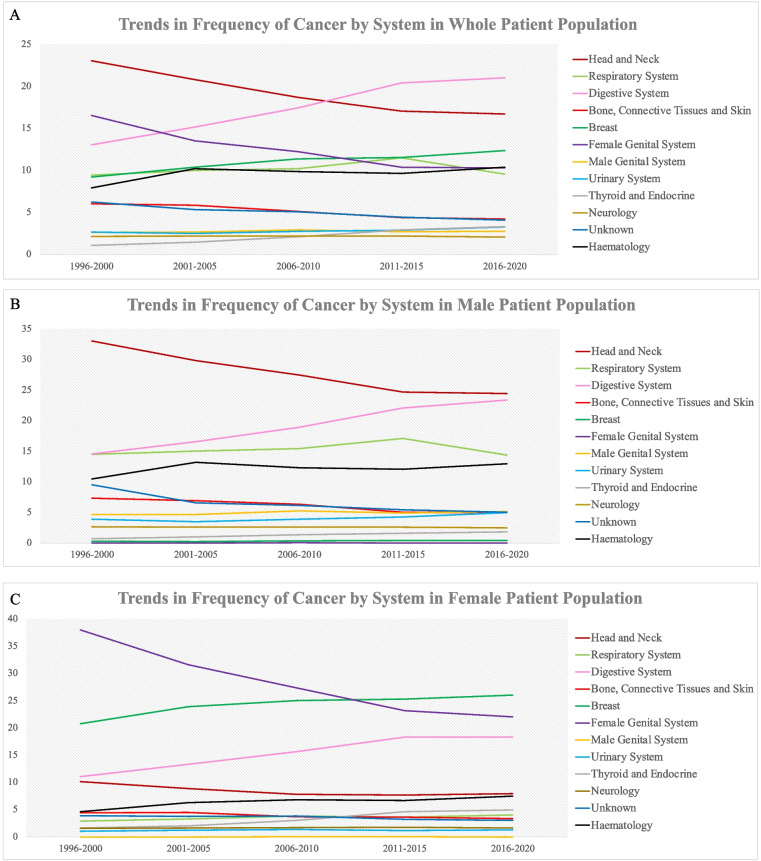
Graph showing trends in the frequency of cancers by the system in the whole patient population (**A**), male patient population (**B**) and female patient population (**C**). Data is presented as a percentage of the total number of malignant cases for that time period

Considering cancers by system showed that the system most commonly affected by cancer in the whole population in the most recent period was the digestive system, followed by the head and neck, breast, haematological and female genital system. In males, head and neck cancers were the most common cancers during the same time period, followed by digestive system cancers, respiratory system cancers, haematological cancers and male genital system cancers. In contrast, in females, we found that the most common system affected was the breast, followed by the female genital system, digestive system, head and neck and haematological system (Fig. 2 and Supplementary Table 2).

**Fig. 3:**
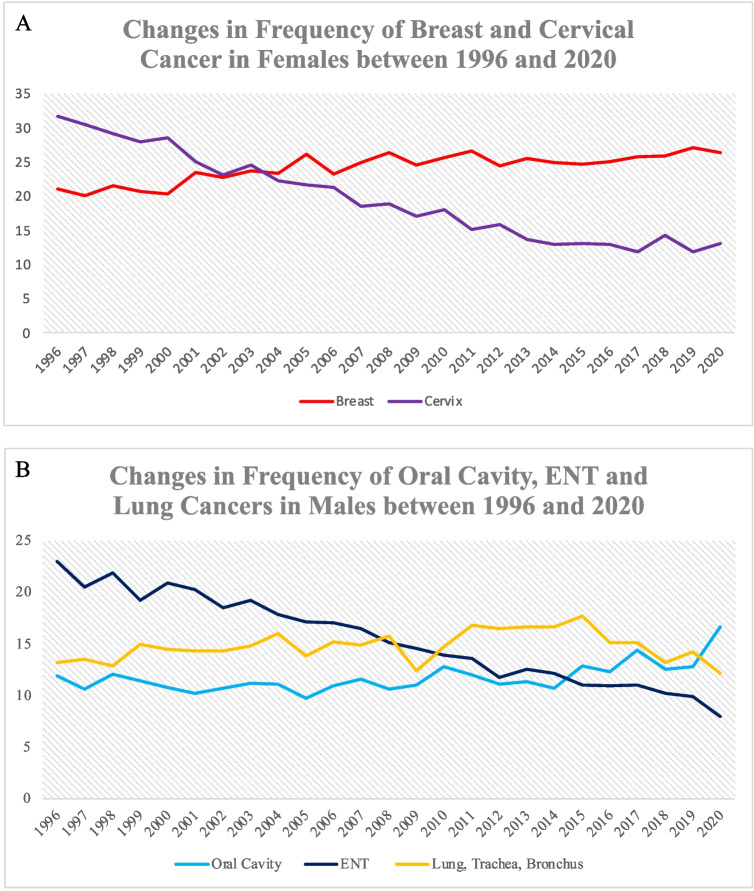
Graph showing changes in the frequency of breast and cervical cancers in females (**A**) and cancers of the oral cavity, ear, nose and throat regions and lung in males (**B**). Data is shown as a percentage of the total number of malignant cases reported for that year

Graphical analysis revealed an increase in the frequency of breast cancer in West Bengal since 1996. Additionally, there has been a reduction in the frequency of cervical cancer in this time period which fell from being the most common cancer in females in the period of 2003–2004 when it was overtaken by breast cancer (Fig. 3). Furthermore, the relative frequency of cancers of the ear, nose and throat regions decreased whilst those of oral cavity and lung cancers increased (Fig. 3).

**Fig. 4:**
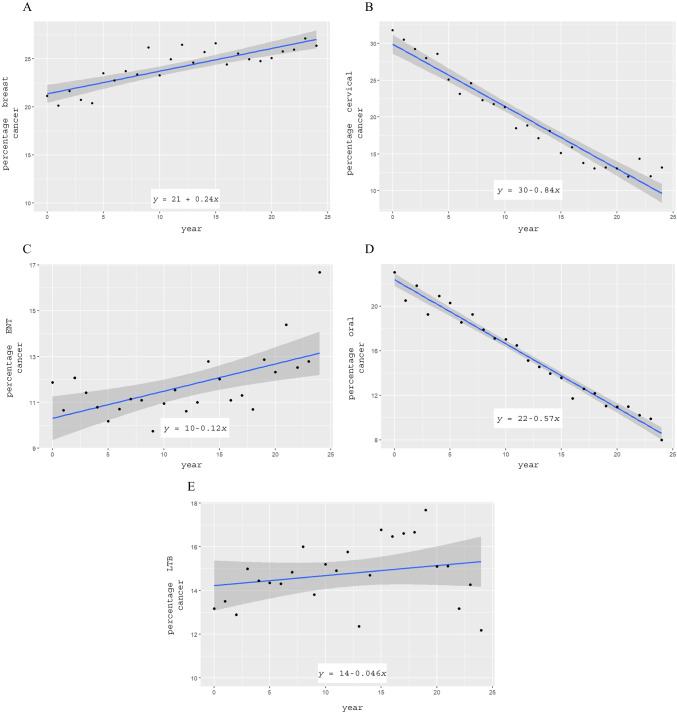
Linear trend analysis showing linear trends fitted to cases of breast cancer (**A**) and cervical cancer (**B**) amongst women and cancers of the ear, nose and throat regions (**C**); oral (**D**); and lung, trachea and bronchus cancers (**E**) amongst males

To test if there is a significant increase or decrease in the frequency of these cancers, we conducted a linear trend analysis as detailed in the methods. The results are summarised in Table 1 and Fig. 4.

**Table 1:** Summarised results of linear trend analyses of the trends of cases of breast and cervical cancers amongst women and cancers of the ear, nose and throat regions amongst males

Patient group	Cancer type	Trend equation	*R*^2^ value	*b* value	*p* value	Inference
Females	Breast	$y=21.36093+0.23674t$	0.6995	0.23674	< 0.0001	Increasing trend
	Cervical	$y=29.89149-0.84395t$	0.9398	− 0.84395	< 0.0001	Decreasing trend
Males	Ear, nose and throat	$y=22.37282-0.57493t$	0.9471	− 0.57439	< 0.0001	Decreasing trend

The mathematical model performed effectively for female breast cancer, cervical cancer and male cancers of the ear, nose and throat regions, demonstrating significant R-squared values. The results revealed increasing trends in female breast cancer, while female cervical cancer and cancers of the ear, nose and throat regions in males showed decreasing trends (Table 1).

In contrast, oral, lung, trachea and bronchus cancers did not fit the model well as evidenced by the low calculated *R*-squared values. For these cancers, a Mann–Kendall test was used to determine the presence of a monotonic upward or downward trend of the variable of interest over time, which may or may not be linear. This revealed the presence of a significant non-linear upward trend for oral cancers (τ  = 0.387, *p* = 0.0072452) and no significant trend (*p* = 0.15426) for lung, trachea and bronchus cancer cases.

## Discussion

The latest GLOBOCAN report estimates an incidence rate of 19.96 million new cases and 9.74 million deaths from cancer in the year 2022 [25]. India ranked third in number of cases, closely following China and the USA [26]. In recent years, the burden of cancer in India has increased greatly and is projected to increase further in the coming years [27, 28]. This increase is thought to result from a multitude of factors including increasing longevity of the population, smoking and lifestyle changes and increasing obesity [4, 10]. In addition, enhanced diagnostic capabilities and improved data collection have also led to significant changes [4].

Looking broadly at cancer frequency, our data showed similar trends to that of the rest of India. A study using data from the National Cancer Registry Programme between 2012 and 2016 to project trends for 2020 found that the projected most common sites for cancer during the period were breast, lung, mouth, cervix uteri and tongue amongst the whole population [6]. Amongst males, the most common sites were the lung, mouth, prostate, tongue and stomach whilst amongst females the most common sites were the breast, cervix uteri, ovary, corpus uteri and lung [6]. Furthermore, the NCRP 2023 report estimated that in 2025, the number of new cancer cases will be 1,569,793 with the most common cancer sites predicted as female breast (238,908), lung (111,328) and mouth (90,060) [3]. The report also indicated that, in 2021, the top ten cancers responsible for the highest number of disability-adjusted life years (DALYs) were lung, breast, oral cavity, cervical, leukaemia, ovarian, oesophageal, stomach, colorectal and lymphoma and multiple myeloma [3].

Our data showed breast cancer to be the most common cancer diagnosed at the hospital. Breast cancer has seen a consistent increase in incidence since 1996, from being the third most common cancer diagnosed after the cervix and cancers of the ear, nose and throat region which had since declined. This is consistent with findings from various other studies [2–4, 9, 10]. It is thought that these changes have been caused by urbanisation and modernisation of India. For example, changes to more westernised, fatty diets, as well as changes in patterns of childbearing and breastfeeding where women are more likely to have fewer children or have children at a later age and are less likely to breastfeed, are all risk factors that may contribute to the increased incidence of breast cancer in the population [4, 10]. In addition, with increasing population size and longer life spans, Indian people are increasingly more prone to developing lifestyle-related cancers, such as breast cancer [4]. This change in the frequency of breast cancer diagnoses suggests a potential role for screening in the Indian population to detect breast cancers earlier and reduce morbidity and mortality.

Similar to previous studies, we found that this increase in breast cancer was juxtaposed with a decrease in the frequency of cervical cancers amongst the patient population [2–4, 17]. In contrast to breast cancer, it is thought that improvements in education, reductions in child marriage, better personal hygiene and family planning have caused a decline in cervical cancer. For example, between 2001 and 2021, child marriage fell from 49% in 2001 to 23% in 2021 and is continuing to fall [29]. Despite these reductions, cervical cancer remains the eighth most common cancer amongst our patient cohort in the most recent period (2016–2020) as well as the second most common cancer amongst women during this period. Interestingly, the rates of cervical cancer have been seen to be low amongst Muslims [30], indicating the potential role of male circumcision as a primary prevention measure for cervical cancer, by reducing the risk of transmission of the human papillomavirus, the most common cause of cervical cancer [31]. Similarly, it also highlights the benefit of HPV screening to reduce transmission of the virus within the population [32].

Our study also found a high frequency of tobacco-related cancers, namely oral cavity, oropharyngeal and laryngeal cancers and lung, trachea and bronchus cancers [33]. This was particularly seen amongst males where they were the top three most common sites of cancers. Similar findings have been noted in other studies, and rates have further been seen to be increasing in various states across India, including in Chennai and Delhi for lung cancers and in Bhopal and Mumbai where increases in mouth cancers were seen [2, 34]. The high frequency of tobacco-related cancers highlights the importance of tobacco control and education on the risks of tobacco and paan use in reducing cancer rates, as well as many other chronic diseases, in India [4]. Interestingly, we observed a decrease in the incidence of cancer of the nasal cavity. This likely reflects a change in tobacco habits from chewing or nasal instillation to smoking [14]. Notably, a study carried out in Mumbai found a decrease in oral cancers which they attributed to reductions in paan and tobacco use [14], highlighting how regional differences may affect cancer incidence across India and the importance of having reliable data to guide policy in each locality.

In addition to tobacco use, air pollution has also been shown to be a leading risk factor for lung cancer [33], and studies have shown that lung cancers are more common in metropolitan regions [3] where it is likely that air pollution levels are higher. At the end of our time period, we found that the frequency of lung cancers appeared to be declining whilst the frequency of oral cavity cancers appeared to be increasing. The reasons for this remain unclear but one hypothesis is that, whilst the diagnosis of most cancers including oral cavity cancers has greatly improved during the study period, lung cancers are still often diagnosed in the advanced stages of the disease [34] leading to a selection bias which may explain this observed trend.

When looking at cancers by the system, we found an overall increase in digestive system cancers amongst the patient population at our institute. This included an increase in the incidence of stomach cancers; duodenal cancers; peritoneum, colon, rectum and anal canal cancers; and liver and gallbladder cancers. In contrast, the rates of oesophageal and pancreatic cancers were observed to be decreasing across the time period studied. Increases in digestive system cancers, particularly colon cancers, in India, have been well noted and are likely due to changes to westernised diets and lifestyles as a result of economic growth in India [8]. Some previous studies, however, have shown a decreasing incidence of stomach cancers [2], once again highlighting the heterogeneity that exists in cancer incidence between different parts of India. This may be due to differing cultural factors, lifestyle choices, geography and pollution levels as well as socioeconomic factors, all of which vary greatly across India.

The strength of this study is that it includes data from more than 135,000 cancer patients over a period of 25 years. During this 25-year period, major changes in economic and lifestyle factors have taken place in India as well as developments in all aspects of cancer management including imaging, detection, staging, therapeutic approaches and prognosis. All of these changes have led to significant changes in cancer epidemiology over this time period. In addition, the hospital at which the study was based caters to a large cross-section of patients from lower socio-economic backgrounds to the more affluent class. It serves people from nearby four states and the neighbouring countries of Bangladesh and Nepal. This diverse patient population enhances the study’s potential to accurately reflect changes in cancer incidence patterns in this region of India. The large sample size further strengthens the statistical analysis, increasing the reliability and relevance of our findings.

A major limitation of our study was that it involved data from a single institution, which may limit the generalisability of the findings. Whilst the institution serves a large and diverse population, the potential for inherent biases remains, and we hope that future work will involve a collaborative effort between major cancer centres in the region to build on this work. Furthermore, the retrospective nature of the data introduces additional constraints, including variations in diagnostic modalities, staging systems and levels of public awareness over the study period. These factors may contribute to biases or lead to the appearance of misleading trends that do not accurately reflect broader epidemiological patterns.

In conclusion, our study has revealed important trends in cancer frequency amongst the population in West Bengal and its surrounding areas. We saw significant increases in breast cancers amongst females juxtaposed with decreases in cervical cancers in line with that seen by other studies carried out in different regions of India. Furthermore, we identified a significant increase in oral cavity cancers and a decrease in oropharyngeal cancers amongst males. Increasing trends were noted in the frequencies of lung, trachea, bronchus and digestive system cancers, although these trends were not found to be statistically significant. Further studies using data from more hospitals in the region are required to build on this work and produce reliable estimates of the frequency and incidence of different cancers in West Bengal.

## Supplementary Information

Below is the link to the electronic supplementary material.

Supplementary file1 (DOCX 158 KB)

## Data Availability

All data supporting the findings of this study are available within the paper and its Supplementary Information.
