# Supplementary material for: Cancer Trends in West Bengal Over 25 Years: A Comprehensive Single-Centre Study
Source: Indian J Surg Oncol. 2025 Mar 1;16(6):1553–62. doi: 10.1007/s13193-025-02252-5 (PMC12708446; doi:10.1007/s13193-025-02252-5)
Supplement: Supplementary file 1 — Supplementary file1 (DOCX 158 KB) [file 13193_2025_2252_MOESM1_ESM.docx]

| **Population** | | **1996-2000** | **2001-2005** | **2006-2010** | **2011-2015** | **2016-2020** | **Overall** |
| --- | --- | --- | --- | --- | --- | --- | --- |
| **All** | **1** | ENT (13.5) | ENT (11.9) | Breast (11.4) | Breast (11.5) | Breast (12.3) | Breast (11.0) |
|  | **2** | Cervix (12.9) | Breast (10.4) | Lung, Trachea, Bronchus (9.6) | Lung, Trachea, Bronchus (11.3) | Oral Cavity (9.8) | Lung, Trachea, Bronchus (9.9) |
|  | **3** | Breast (9.2) | Cervix (10.0) | ENT (9.4) | Oral Cavity (8.6) | Lung, Trachea, Bronchus (9.4) | ENT (9.5) |
|  | **4** | Oral Cavity (9.1) | Lung, Trachea, Bronchus (9.7) | Oral Cavity (8.6) | ENT (7.7) | Liver and Gallbladder (6.4) | Oral Cavity (8.9) |
|  | **5** | Lung, Trachea, Bronchus (8.9) | Oral Cavity (8.4) | Cervix (8.3) | Liver and Gallbladder (6.8) | Haematopoietic System (6.3) | Cervix (8.5) |
|  | **6** | Secondary Malignant Neoplasm of Unknown Primary (5.9) | Haematopoietic System (5.5) | Haematopoietic System (5.7) | Cervix (6.3) | ENT (6.4) | Haematopoietic System (5.4) |
|  | **7** | Bone Connective Tissues (3.9) | Secondary Malignant Neoplasm of Unknown Primary (5.2) | Liver and Gallbladder (5.4) | Haematopoietic System (5.7) | Peritoneum, Colon, Rectum, Anal Canal (6.2) | Liver and Gallbladder (5.4) |
|  | **8** | Oesophagus (3.7) | Liver and Gallbladder (4.4) | Peritoneum, Colon, Rectum, Anal Canal (4.8) | Peritoneum, Colon, Rectum, Anal Canal (5.2) | Cervix (6.0) | Peritoneum, Colon, Rectum, Anal Canal (4.8) |
|  | **9** | Haematopoietic System (3.5) | Peritoneum, Colon, Rectum, Anal Canal (3.9) | Secondary Malignant Neoplasm of Unknown Primary (4.1) | Secondary Malignant Neoplasm of Unknown Primary (4.0) | Stomach (3.8) | Secondary Malignant Neoplasm of Unknown Primary (4.5) |
|  | **10** | Liver and Gallbladder (3.5) | Bone Connective Tissues (3.9) | Lymphatic System (excluding Hodgkin's) (3.4) | Stomach (3.8) | Secondary Malignant Neoplasm of Unknown Primary (3.7) | Bone Connective Tissues (3.4) |
| **Male** | **1** | ENT (21.1) | ENT (18.6) | ENT (15.3) | Lung, Trachea, Bronchus (16.9) | Lung, Trachea, Bronchus (14.2) | ENT (15.2) |
|  | **2** | Lung, Trachea, Bronchus (13.8) | Lung, Trachea, Bronchus (14.6) | Lung, Trachea, Bronchus (14.5) | ENT (12.2) | Oral Cavity (13.4) | Lung, Trachea, Bronchus (14.9) |
|  | **3** | Oral Cavity (11.4) | Oral Cavity (10.6) | Oral Cavity (11.4) | Oral Cavity (11.6) | ENT (10.3) | Oral Cavity (11.7) |
|  | **4** | Secondary Malignant Neoplasm of Unknown Primary (7.7) | Haematopoietic System (6.8) | Haematopoietic System (6.8) | Haematopoietic System (6.8) | Haematopoietic System (7.7) | Haematopoietic System (6.6) |
|  | **5** | Bone Connective Tissues (4.7) | Secondary Malignant Neoplasm of Unknown Primary (6.5) | Peritoneum, Colon, Rectum, Anal Canal (5.7) | Peritoneum, Colon, Rectum, Anal Canal (6.0) | Peritoneum, Colon, Rectum, Anal Canal (7.1) | Secondary Malignant Neoplasm of Unknown Primary (5.6) |
|  | **6** | Haematopoietic System (4.5) | Lymphatic System (excluding Hodgkin's) (4.8) | Secondary Malignant Neoplasm of Unknown Primary (5.0) | Liver and Gallbladder (5.0) | Liver and Gallbladder (5.3) | Peritoneum, Colon, Rectum, Anal Canal (5.5) |
|  | **7** | Oesophagus (4.5) | Bone Connective Tissues (4.5) | Lymphatic System (excluding Hodgkin's) (4.4) | Secondary Malignant Neoplasm of Unknown Primary (4.9) | Stomach (4.7) | Lymphatic System (excluding Hodgkin's) (4.4) |
|  | **8** | Lymphatic System (excluding Hodgkin's) (4.3) | Peritoneum, Colon, Rectum, Anal Canal (4.5) | Oesophagus (4.1) | Stomach (4.8) | Secondary Malignant Neoplasm of Unknown Primary (4.5) | Oesophagus (4.1) |
|  | **9** | Peritoneum, Colon, Rectum, Anal Canal (4.2) | Oesophagus (4.4) | Bone Connective Tissues (4.1) | Lymphatic System (excluding Hodgkin's) (4.1) | Lymphatic System (excluding Hodgkin's) (4.2) | Liver and Gallbladder (4.1) |
|  | **10** | Liver and Gallbladder (2.8) | Stomach (3.8) | Stomach (4.0) | Oesophagus (4.1) | Oesophagus (3.7) | Stomach (4.1) |
| **Female** | **1** | Cervix (29.6) | Breast (23.9) | Breast (25.1) | Breast (25.3) | Breast (26.0) | Breast (24.4) |
|  | **2** | Breast (20.8) | Cervix (23.4) | Cervix (18.7) | Cervix (14.2) | Cervix (12.9) | Cervix (19.1) |
|  | **3** | Oral Cavity (6.1) | Liver and Gallbladder (5.9) | Liver and Gallbladder (7.5) | Liver and Gallbladder (8.9) | Liver and Gallbladder (7.7) | Liver and Gallbladder (7.0) |
|  | **4** | Ovary and Fallopian Tubes (4.7) | Oral Cavity (5.5) | Oral Cavity (5.2) | Ovary and Fallopian Tubes (5.1) | Oral Cavity (5.6) | Oral Cavity (5.4) |
|  | **5** | Liver and Gallbladder (4.4) | Ovary and Fallopian Tubes (4.7) | Ovary and Fallopian Tubes (5.0) | Oral Cavity (4.9) | Ovary and Fallopian Tubes (5.4) | Ovary and Fallopian Tubes (5.0) |
|  | **6** | Secondary Malignant Neoplasm of Unknown Primary (3.6) | Haematopoietic System (3.8) | Haematopoietic System (4.3) | Thyroid (4.6) | Peritoneum, Colon, Rectum, Anal Canal (5.2) | Haematopoietic System (4.0) |
|  | **7** | ENT (3.6) | v (3.6) | Peritoneum, Colon, Rectum, Anal Canal (3.7) | Lung, Trachea, Bronchus (4.5) | Thyroid (5.0) | Peritoneum, Colon, Rectum, Anal Canal (3.8) |
|  | **8** | Bone Connective Tissues (3.0) | Lung, Trachea, Bronchus (3.2) | Lung, Trachea, Bronchus (3.5) | Haematopoietic System (4.3) | Haematopoietic System (4.8) | Lung, Trachea, Bronchus (3.6) |
|  | **9** | Oesophagus (2.6) | Peritoneum, Colon, Rectum, Anal Canal (3.1) | Secondary Malignant Neoplasm of Unknown Primary (3.1) | Peritoneum, Colon, Rectum, Anal Canal (4.2) | Lung, Trachea, Bronchus (3.9) | Thyroid (3.4) |
|  | **10** | Lung, Trachea, Bronchus (2.6) | Bone Connective Tissues (3.0) | Thyroid (3.0) | Secondary Malignant Neoplasm of Unknown Primary (3.0) | Stomach (2.8) | Secondary Malignant Neoplasm of Unknown Primary (3.2) |

**Supplementary Table 1:** Table showing the top ten most common cancers by percentage in the overall patient population, male patients, and female patients in 5-year intervals from 1996 to 2020, and for the entire period. Data is presented as a percentage of the total number of malignant cases for that time period.

| **Population** | | **1996-2000** | **2001-2005** | **2006-2010** | **2011-2015** | **2016-2020** | **Overall** |
| --- | --- | --- | --- | --- | --- | --- | --- |
| **All** | **1** | Head and Neck (23.0) | Head and Neck (20.8) | Head and Neck (18.7) | Digestive System (20.4) | Digestive System (21.0) | Head and Neck (19.1) |
|  | **2** | Female Genital System (16.5) | Digestive System (15.1) | Digestive System (17.5) | Head and Neck (17.0) | Head and Neck (16.7) | Digestive System (17.7) |
|  | **3** | Digestive System (13.0) | Female Genital System (13.5) | Female Genital System (12.2) | Breast (11.5) | Breast (12.3) | Female Genital System (12.4) |
|  | **4** | Respiratory System (9.4) | Breast (10.4) | Breast (11.4) | Respiratory System (11.5) | Haematology (10.4) | Breast (11.0) |
|  | **5** | Breast (9.2) | Haematology (10.2) | Respiratory System (10.2) | Female Genital System (10.4) | Female Genital System (10.3) | Respiratory System (10.2) |
|  | **6** | Haematology (7.9) | Respiratory System (10.0) | Haematology (9.9) | Haematology (9.6) | Respiratory System (9.5) | Haematology (9.6) |
|  | **7** | Unknown (6.3) | Bone, Connective Tissues and Skin (5.8) | Bone, Connective Tissues and Skin (5.1) | Unknown (4.4) | Bone, Connective Tissues and Skin (4.2) | Bone, Connective Tissues and Skin (5.1) |
|  | **8** | Bone, Connective Tissues and Skin (6.0) | Unknown (5.3) | Unknown (5.1) | Bone, Connective Tissues and Skin (4.4) | Unknown (4.1) | Unknown (5.0) |
|  | **9** | Urinary System (2.6) | Male Genital System (2.7) | Male Genital System (2.9) | Thyroid and Endocrine (2.9) | Thyroid and Endocrine (3.3) | Urinary System (2.8) |
|  | **10** | Male Genital System (2.6) | Urinary System (2.5) | Urinary System (2.8) | Urinary System (2.9) | Urinary System (3.3) | Male Genital System (2.7) |
| **Male** | **1** | Head and Neck (33.0) | Head and Neck (29.8) | Head and Neck (27.5) | Head and Neck (24.7) | Head and Neck (24.4) | Head and Neck (27.7) |
|  | **2** | Digestive System (14.6) | Digestive System (16.5) | Digestive System (18.9) | Digestive System (22.1) | Digestive System (23.4) | Digestive System (19.3) |
|  | **3** | Respiratory System (14.5) | Respiratory System (15.0) | Respiratory System (15.4) | Respiratory System (17.1) | Respiratory System (14.4) | Respiratory System (15.3) |
|  | **4** | Haematology (10.5) | Haematology (13.2) | Haematology (12.3) | Haematology (12.0) | Haematology (12.9) | Haematology (12.2) |
|  | **5** | Unknown (9.5) | Bone, Connective Tissues and Skin (6.9) | Bone, Connective Tissues and Skin (6.3) | Unknown (5.4) | Male Genital System (5.1) | Unknown (6.5) |
|  | **6** | Bone, Connective Tissues and Skin (7.3) | Unknown (6.6) | Unknown (6.2) | Bone, Connective Tissues and Skin (5.0) | Unknown (5.0) | Bone, Connective Tissues and Skin (6.0) |
|  | **7** | Male Genital System (4.6) | Male Genital System (4.7) | Male Genital System (5.3) | Male Genital System (4.9) | Urinary System (5.0) | Male Genital System (4.9) |
|  | **8** | Urinary System (3.9) | Urinary System (3.5) | Urinary System (3.9) | Urinary System (4.3) | Bone, Connective Tissues and Skin (5.0) | Urinary System (4.1) |
|  | **9** | Neurology (2.6) | Neurology (2.6) | Neurology (2.6) | Neurology (2.6) | Neurology (2.5) | Neurology (2.6) |
|  | **10** | Thyroid and Endocrine (0.7) | Thyroid and Endocrine (1.0) | Thyroid and Endocrine (1.3) | Thyroid and Endocrine (1.6) | Thyroid and Endocrine (1.8) | Thyroid and Endocrine (1.3) |
| **Female** | **1** | Female Genital System (38.0) | Female Genital System (31.6) | Female Genital System (27.3) | Breast (25.3) | Breast (26.0) | Female Genital System (27.8) |
|  | **2** | Breast (20.8) | Breast (23.9) | Breast (25.1) | Female Genital System (23.1) | Female Genital System (22.0) | Breast (24.4) |
|  | **3** | Digestive System (11.0) | Digestive System (13.3) | Digestive System (15.6) | Digestive System (18.3) | Digestive System (18.3) | Digestive System (15.6) |
|  | **4** | Head and Neck (10.2) | Head and Neck (8.9) | Head and Neck (7.8) | Head and Neck (7.6) | Head and Neck (7.9) | Head and Neck (8.4) |
|  | **5** | Haematology (4.6) | Haematology (6.3) | Haematology (6.8) | Haematology (6.7) | Haematology (7.5) | Haematology (6.5) |
|  | **6** | Bone, Connective Tissues and Skin (4.4) | Bone, Connective Tissues and Skin (4.5) | Respiratory System (3.8) | Thyroid and Endocrine (4.6) | Thyroid and Endocrine (5.0) | Bone, Connective Tissues and Skin (3.9) |
|  | **7** | Unknown (3.9) | Unknown (3.7) | Unknown (3.8) | Respiratory System (3.6) | Respiratory System (4.0) | Respiratory System (3.6) |
|  | **8** | Respiratory System (2.9) | Respiratory System (3.3) | Bone, Connective Tissues and Skin (3.7) | Bone, Connective Tissues and Skin (3.6) | Bone, Connective Tissues and Skin (3.4) | Unknown (3.5) |
|  | **9** | Thyroid and Endocrine (1.6) | Thyroid and Endocrine (2.0) | Thyroid and Endocrine (3.0) | Unknown (3.2) | Unknown (3.0) | Thyroid and Endocrine (3.4) |
|  | **10** | Neurology (1.6) | Neurology (1.6) | Neurology (1.7) | Neurology (1.7) | Neurology (1.6) | Neurology (1.6) |

**Supplementary Table 2:** Table showing trends in frequency of cancers by system in the overall patient population, male patients, and female patients in 5-year intervals from 1996 to 2020, and for the entire period. Data is presented as a percentage of the total number of malignant cases for that time period.

**Supplementary Fig.1:** Graph showing trends in malignant, non-malignant and un-diagnosed and total cancer cases in the whole patient population (A), male population (B) and female population (C).

**Supplementary Information: Mathematical Model Used for Linear Trend Analysis**

We used the following mathematical model for linear trend analysis:

$\boldsymbol{y=a+bt}$,

y = percentage of cases over the period 1996-2020

t = time in years, starting from 1996

constants a & b = estimated using method of least squares; a positive value b indicates increasing trend and a negative value indicates decreasing trend; values near zero indicate no overall trend.
